# A young patient with Graves' disease presenting with a triad of heart failure, pancytopenia, and jaundice: A case report

**DOI:** 10.1002/ccr3.8698

**Published:** 2024-04-24

**Authors:** Mohamed Kamal Sabra, Anwar Joudeh, Mahmoud Al Niserat, Riyadh Hammamy

**Affiliations:** ^1^ Department of Internal Medicine Al‐Khor Hospital, Hamad Medical Corporation Doha Qatar; ^2^ Internal Medicine, Collage of Medicine University of Qatar Doha Qatar; ^3^ Department of Medical Education Hamad Medical Corporation Doha Qatar

**Keywords:** cardiomyopathy, Grave's disease, jaundice, pancytopenia

## Abstract

**Key Clinical Message:**

Graves's disease must be treated promptly to avoid serious sequelae such as cardiomyopathy, liver injury, and pancytopenia. Early initiation of antithyroid medications and beta blockers could not be overrated even in the presence of these complications.

**Abstract:**

Graves' disease causes a large spectrum of clinical manifestations. Delayed diagnosis and management of Graves' disease could lead to serious systemic sequelae. We describe a case of a young man who presented with progressive cough, increased abdominal girth and ankle swelling for a few months. On examination, he had jaundice, bilateral exophthalmos, diffuse goiter, ascites, and significant lower limb edema. Laboratory investigations showed increased Thyroxin level with a suppressed thyroid stimulating hormone and positive anti‐thyrotropin receptor antibodies. Also, the patient had pancytopenia, coagulopathy and cholestatic pattern of elevated liver enzymes. Echocardiography demonstrated mildly reduced left ventricular function with diastolic dysfunction, but electrocardiogram did not show atrial fibrillation. Despite the concerns about using antithyroid medications in patients with impaired liver function tests and pancytopenia, the patient improved dramatically without worsening of his hematological or biochemical parameters. Early initiation of antithyroid medications and beta blockers is essential for patients who are newly diagnosed with hyperthyroidism.

## 1 INTRODUCTION

Graves' disease often presents with florid hypermetabolic and hyperdynamic manifestations including heat intolerance, palpitations, hyperphagia, and weight loss. Symptoms are largely related to the high circulatory thyroid hormone levels, but it is also uniquely associated with several disorders that are not directly attributed to thyroid hormone excess such as Graves' disease ophthalmopathy and dermopathy. Systemic complications of hyperthyroidism such as heart failure, cholestatic jaundice and pancytopenia are rarely reported at presentation.^1^ Only a minority of patients who were newly diagnosed with hyperthyroidism had heart failure at presentation,^2^ while the co‐occurrence of pancytopenia with Graves' disease was only reported through a few case reports.^3^ Nevertheless, abnormal levels of liver enzymes are not uncommon at presentation.^4^ Herein, we describe a case of a previously healthy young man who presented with fever, cough and lower limb edema and was later diagnosed to have Graves' disease‐related heart failure with congestive hepatopathy and pancytopenia. The patient improved clinically and biochemically with carbimazole and propranolol therapy.

## 2 CASE REPORT

### 2.1 Case history and examination

A 29‐year‐old Nepalese male presented to hospital with fever and cough for a few days. He also complained of progressive lower limb swelling, increased abdominal girth, and yellowish discoloration of the sclera over the past few months. No previous history of chronic medical illness and he was not using any regular medications. He denied substance abuse, smoking, or alcohol intake. His family history was also negative for hematological disorders, autoimmune diseases, or contagious infections.

On examination, he was pale and jaundiced with mild bilateral exophthalmos and diffuse goiter. His blood pressure was 123/72, and his heart rate was 120 beats/min. His body mass index was 22.5 kg/m^2^. He had raised jugular venous pressure and reduced breath sounds with dullness over the right lower chest. The abdomen was distended with shifting dullness, and the liver edge was palpable 2 cm below the costal margin. He had significant bilateral pitting edema up to the mid‐shin of the tibia. Neurological examination was normal except for a bilateral postural hand tremor.

### 2.2 Differential diagnosis, investigations, and treatment

The laboratory investigations were significant for pancytopenia, prolonged prothrombin and partial thromboplastin times, direct hyperbilirubinemia, and mildly elevated transaminases and alkaline phosphatase levels in a cholestatic pattern with an *R* value of 0.9 (*R* ≥ 5: hepatocellular injury, *R* > 2 to <5: mixed pattern, *R* ≤ 2: cholestatic injury^5^) as shown in Table 1. Thyroid function tests were consistent with primary hyperthyroidism with positive thyrotropin receptor antibody (TRAb) and anti‐thyroid peroxidase antibody titers. Viral hepatitis serology, autoimmune hepatitis antibodies and celiac serology were all negative. We also investigated the patients for other causes of pancytopenia such as infections (Human Immunodeniecny Virus (HIV) infection, leishmaniasis, tuberculosis), nutritional deficiencies (serum vitamin B12 and folate levels), autoimmune diseases (Antinuclear Antibodies (ANA) and other autoantibodies) and they were all negative. The only positive finding was low iron saturation which contributed to the anemia but cannot explain the trilineage suppression (Table 1). However, we did not have a baseline blood investigation to compare them to this presentation.

**TABLE 1:** Summary of laboratory investigations at presentation, day 7 and day 21.

Laboratory test	Presentation	Day 7	Day 21	Normal range
Hemoglobin (g/dL)	5.7	8.1	9.5	13–17
White Blood Cell count (*10^3^/μL)	3.3	3.3	3.9	4–10
Absolute Neutrophil Count (*10^3^/μL)	1.3	1.3	1.7	2–7
Platelet (*10^3^/μL)	84	107	146	150–400
Bilirubin (μmol/L)	35 0.0	41.0	36.0	0–21
Direct bilirubin (μmol/L)	31.0	33.0	27.0	0–5
ALT (U/L)	57.0	88.0	23.0	0–41
AST (U/L)	99.0	63.0	27.0	0–40
Alkaline phosphatase (U/L)	200	178	151	40–129
PT (seconds)	20.6	17.5	12.6	9.7–11.8
INR	2.0	1.7	1.2	
PTT (seconds)	41.1	37.0	28.2	24.6–31.2
Pro‐BNP (pg/mL)	961	608	–	<125
TSH (micro‐IU/L)	<0.01	–	<0.01	0.3–4.2
Free T4 (pmol/L [pg/mL])	>100 [65]	–	23.4 [15.1]	11–23.3 [7.1–15.1]
Free T3 (pmol/L[pg/mL])	20.5 [13.3]	–	7.4 [4.8]	3.7–6.7 [2.6–5.1]
Ferritin (μg/L)	43.3			48–420
Transferrin saturation (%)	4.17			15–45
Haptoglobin (mg/dL)	30			30–200
Vitamin B12 (pmol/L)	685			145–596
Retic percentage	2.1			0.5–2.5
Thyrotropin receptor antibody (TRAb) (IU/L)	25.1	–	–	Positive ≥1.75
Anti‐thyroid peroxidase antibody (IU/mL)	436			0–34
Peripheral smear	Hypochromic microcytic RBCs with few oval cells and target cells. Leukopenia with neutropenia plus mild thrombocytopenia			

**FIGURE 1:**
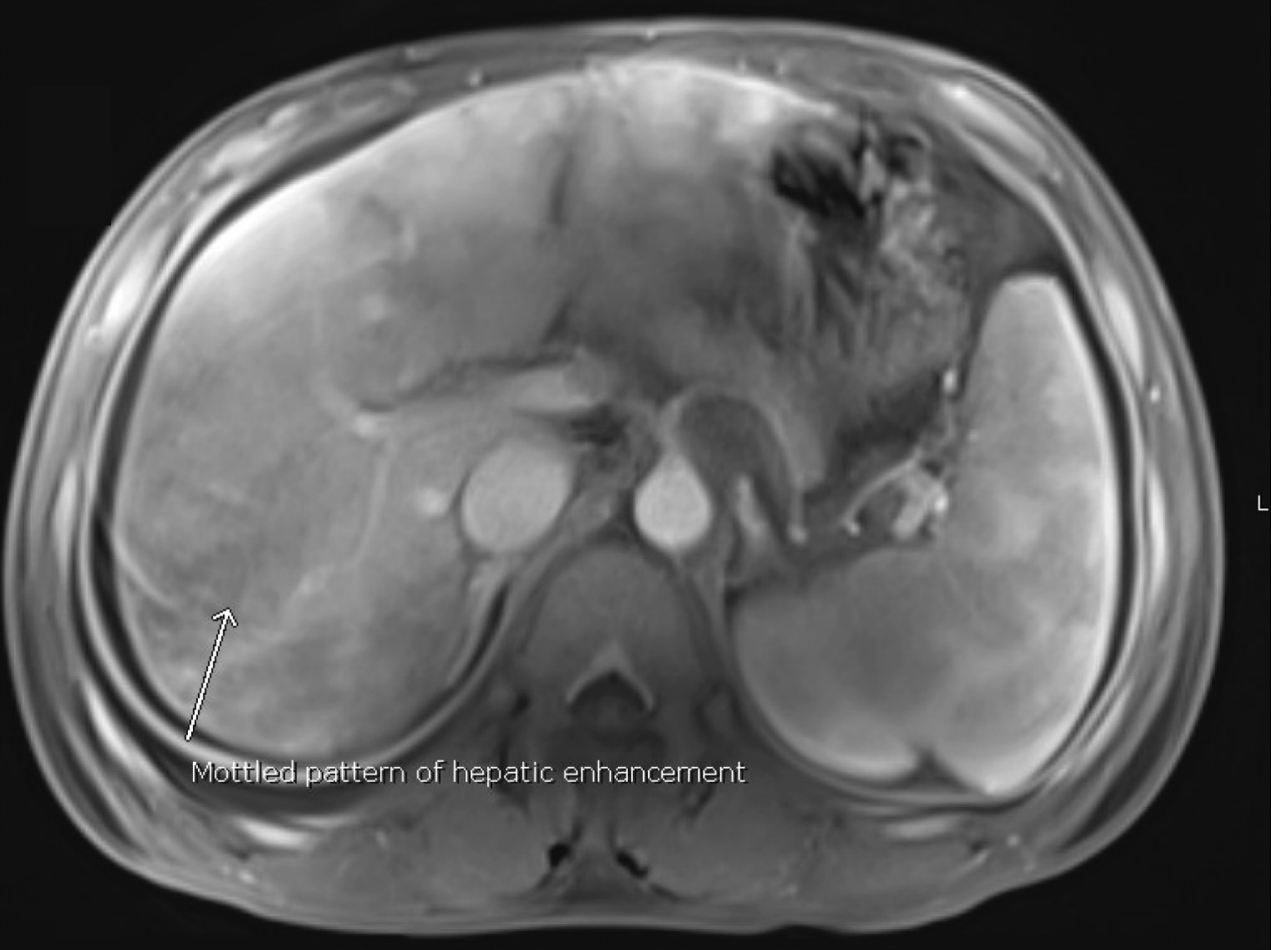
MRI abdomen with intravenous contrast showing mottled appearance of the liver parenchyma.

**FIGURE 2:**
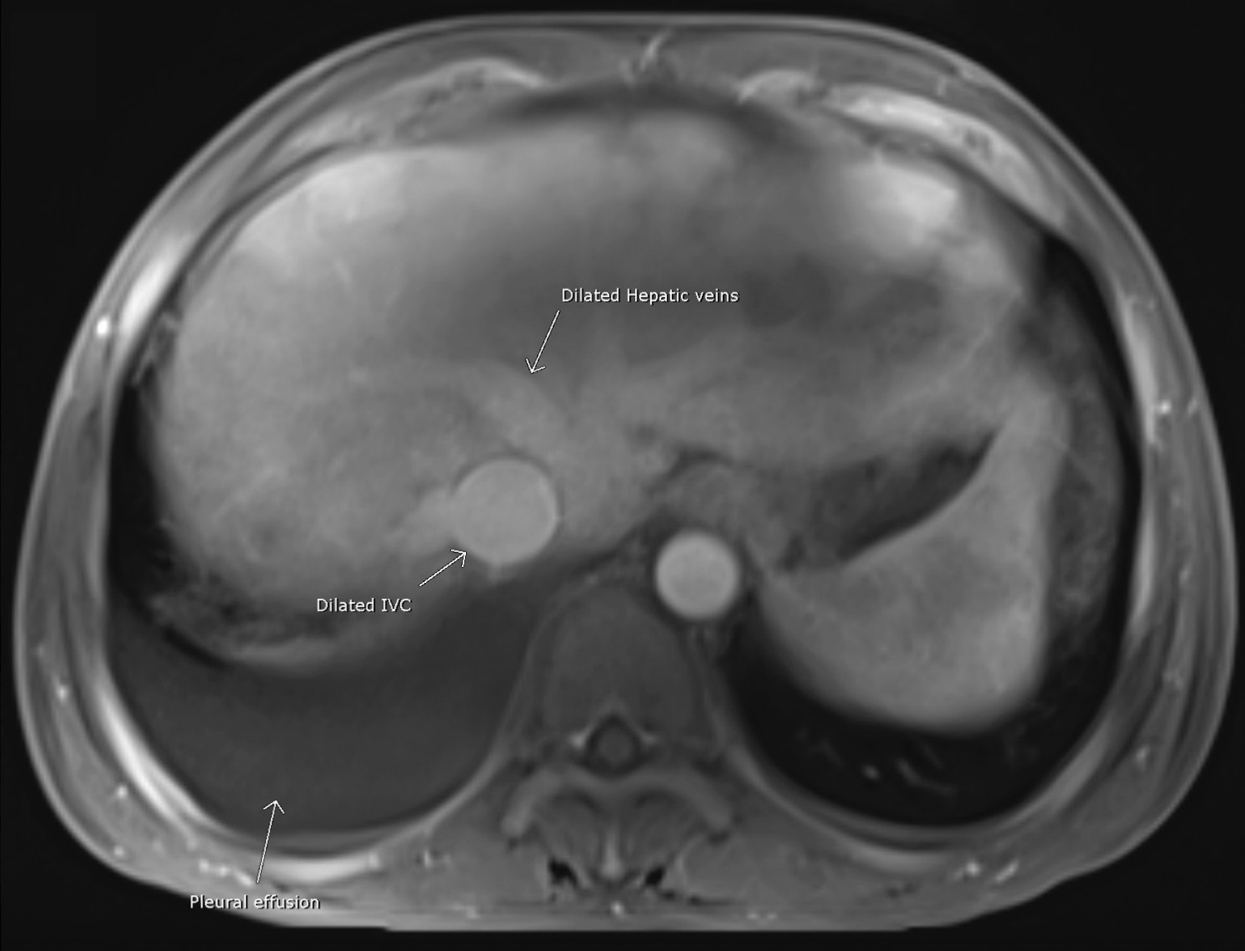
MRI abdomen with intravenous contrast showing a dilated inferior vena cava (IVC) and prominent hepatic veins in addition to right‐sided pleural effusion.

The chest X‐ray showed cardiomegaly with obliteration of the right costophrenic angle while ultrasound imaging of the abdomen revealed mild to moderate ascites, gallbladder calculus and an edematous thickened gallbladder wall of 6 mm. However, sonographic Murphy sign was negative. Magnetic resonance cholangiopancreatography excluded biliary tree obstruction, but it was suggestive for congestive hepatopathy with a mottled appearance of the liver parenchyma (Figure 1) and prominent inferior vena cava and hepatic veins (Figure 2). An echocardiogram showed a mildly reduced left ventricular ejection fraction (43%), mild left ventricular hypertrophy, and grade III diastolic dysfunction. Ultrasound examination of the thyroid gland demonstrated a diffusely enlarged gland with increased vascularity.

### 2.3 Outcome and follow‐up

Subsequently, the patient was diagnosed with primary hyperthyroidism secondary to Graves' disease and we started him on carbimazole (20 mg twice daily), propranolol (20 mg twice daily), and furosemide (40 mg once daily) with a gradual resolution of his symptoms. Repeated laboratory investigations at day 21 showed improvement in the full blood count, coagulation profile and liver enzymes parameters (Table 1). We planned to repeat echocardiography after 2 months of therapy and titrate his medications but the patient traveled back to his home country.

## 3 DISCUSSION

New‐onset heart failure in patients with newly diagnosed hyperthyroidism is not common. A cohort study from China showed that only 6% of the newly diagnosed patients with hyperthyroidism had heart failure at presentation. Unlike this case, the majority were older adults (median age 66 years), with concomitant atrial fibrillation, diabetes mellitus, hypertension, and/or smoking.^2^ It is possible that delayed presentation of our patient and the long‐standing tachycardia contributed to his hyperthyroid cardiomyopathy.^6^ The use of beta blockers and restoration of euthyroid status were associated with marked resolution of heart failure symptoms in previous reports and in this case.^2^

Pancytopenia in this case was likely secondary to Graves' disease as we excluded other potential reasons such as vitamin B12 deficiency, viral infections, leishmaniasis, or autoimmune diseases. We only found low iron saturation but that would not explain the low leukocytes or platelets count. Also, the trilineage hematopoiesis improved after treating hyperthyroidism. Hyperthyroidism is associated with several hematological disorders including neutropenia and normochromic anemia; however, pancytopenia was less frequently reported.^3^, ^7^ A literature review found only 30 cases of concomitant pancytopenia with hyperthyroidism through 2020, 29 cases of them had overt thyrotoxicosis due to Graves' disease, and 90% recovered from pancytopenia with euthyroid status restoration irrespective of the type of treatment.^3^ In this case presentation, the full blood count improved markedly at day 21 although the WBC count was still borderline low. According to the previously mentioned review, the median duration of full recovery of pancytopenia from the time of starting antithyroid medications was 12 weeks in a range of 4 days up to 12 months.^3^ Nevertheless, it must be noted that the few cases with persistent pancytopenia had concomitant untreated vitamin B12 deficiency or aplastic anemia.^3^ Consequently, it is important to exclude other potentially associated autoimmune conditions with Graves' disease that might affect hematopoiesis.

The American Thyroid Association guidelines (2016) recommend reconsidering the use of antithyroid medications in patients with a baseline absolute neutrophil count of less than 1000 cells/microL.^8^ Nevertheless, a more recent systematic review on 1144 patients with untreated hyperthyroidism showed that neutropenia resolved in all patients treated with antithyroid medications without worsening of the baseline neutrophil count or development of agranulocytosis.^9^ Therefore, the decision to start antithyroid medications in patients with neutropenia should be individualized considering the rare occurrence of drug‐induced agranulocytosis and patient's choice of treatment modality.

As shown in this case, untreated hyperthyroidism can be associated with serious impairment of liver function with coagulopathy and ascites. Liver injury in untreated hyperthyroidism is likely multifactorial through direct hepatocyte injury, congestive cardiomyopathy, concomitant autoimmune disease, and/or a previous underlaying liver disease.^10^ In this case, imaging features were consistent with congestive hepatopathy secondary to heart failure. An earlier case series on liver function abnormalities in hyperthyroidism showed that patients with heart failure were more likely to have deeper jaundice, worse coagulopathy, and ascites in comparison to patients without heart failure.^11^ The possibility of concomitant autoimmune hepatic disorders in patients with Graves' disease may present a diagnostic dilemma and treatment challenges.^9^ Despite the various causes for hepatic dysfunction in hyperthyroidism, antithyroid medications seem to have beneficial effects on the liver function tests particularly when transaminases levels are less than five‐fold the upper limit of normal.^4^, ^10^

This case report presented the clinical presentation and successful management of a complicated case of Graves' disease in a young individual. However, our findings are limited by presenting a single case report. Investigating a larger series of patients with Graves' disease is required to identify the entire spectrum of this protean illness. Also, we acknowledge the fact that the short duration of follow up in this case limited further evaluation of his cardiac function and long‐term outcome. However, we highlighted several clinical messages for internists and emergency doctors. As demonstrated in this case, untreated Graves' disease could lead to a number of serious complications including cardiomyopathy, bone marrow suppression and hepatic dysfunction in previously fit young individuals. Therefore, early recognition and initiation of therapy cannot be overrated. Uncontrolled hyperthyroidism is mainly associated with anemia but other lineage of hematopoiesis could be affected as well. Successful management of thyrotoxicosis should lead to reversal of these ominous findings in the absence of other concomitant autoimmune diseases affecting hematopoiesis. The first line intervention for hyperthyroid cardiomyopathy is by suppressing the hyperadrenergic status of patients through early introduction of beta blockers and antithyroid medications. The liver injury observed in Graves' disease is likely multifactorial either directly by thyrotoxicosis‐induced hepatic cell dysfunction or indirectly by the associated congestive heart failure or autoimmune liver diseases.

Treating physicians should not hesitate to carefully introduce beta blockers and antithyroid medications in newly diagnosed patients with hyperthyroidism despite the concerns of pancytopenia, elevated liver enzymes or symptoms of decompensated heart failure. According to our observation and the literature review,^3^, ^4^, ^8^, ^9^ the use of these medications in hyperthyroid‐associated complications is largely safe and effective in reversing these consequences and achieving complete recovery.

## AUTHOR CONTRIBUTIONS

**Mohamed Kamal Sabra:** Conceptualization; investigation; writing – review and editing. **Anwar I Joudeh:** Conceptualization; data curation; formal analysis; writing – original draft; writing – review and editing. **Mahmoud Al Niserat:** Data curation; writing – original draft. **Riyadh Hammamy:** Conceptualization; project administration; supervision; writing – review and editing.

## FUNDING INFORMATION

The authors have not declared a specific grant for this research from any funding agency in the public, commercial or not‐for‐profit sectors. Open Access fee was provided by Qatar National Library.

## CONFLICT OF INTEREST STATEMENT

The authors have no conflict of interest to declare.

## ETHICS STATEMENT

This work was conducted in accordance with the Declaration of Helsinki (1964). This case report was approved by the Institutional Review Board at Hamad Medical Corporation, Doha, Qatar (reference number: MRC‐04‐23‐735).

## CONSENT

Written informed consent was obtained from the patient to publish this report in accordance with the journal's patient consent policy.

## Data Availability

Data can be obtained from the corresponding author upon request.

## References

[1] Smith TJ , Hegedüs L . Graves' disease. N Engl J Med. 2016;375(16):1552‐1565. doi:10.1056/NEJMra1510030 27797318

[2] Siu CW , Yeung CY , Lau CP , Kung AW , Tse HF . Incidence, clinical characteristics and outcome of congestive heart failure as the initial presentation in patients with primary hyperthyroidism. Heart. 2007;93(4):483‐487. doi:10.1136/hrt.2006.100628 17005710 PMC1861478

[3] Scappaticcio L , Bellastella G , Maiorino MI , et al. Graves' hyperthyroidism‐related pancytopenia: a case report with literature review. Hormones (Athens). 2021;20(1):93‐100. doi:10.1007/s42000-020-00227-5 32638234

[4] Scappaticcio L , Longo M , Maiorino MI , et al. Abnormal liver blood tests in patients with hyperthyroidism: systematic review and meta‐analysis. Thyroid. 2021;31(6):884‐894. doi:10.1089/thy.2020.0715 33327837

[5] Chalasani NP , Hayashi PH , Bonkovsky HL , et al. ACG clinical guideline: the diagnosis and management of idiosyncratic drug‐induced liver injury. Am J Gastroenterol. 2014;109(7):950‐967. doi:10.1038/ajg.2014.131 24935270

[6] Klein I , Ojamaa K . Thyroid hormone and the cardiovascular system. N Engl J Med. 2001;344(7):501‐509. doi:10.1056/NEJM200102153440707 11172193

[7] Ross DS , Cooper DS , Mulder JE . Overview of the clinical manifestations of hyperthyroidism in adults. UpToDate. UpToDate; 2023.

[8] Ross DS , Burch HB , Cooper DS , et al. American Thyroid Association guidelines for diagnosis and management of hyperthyroidism and other causes of thyrotoxicosis. Thyroid. 2016;26(10):1343‐1421. doi:10.1089/thy.2016.0229 27521067

[9] Scappaticcio L , Maiorino MI , Maio A , Esposito K , Bellastella G . Neutropenia in patients with hyperthyroidism: systematic review and meta‐analysis. Clin Endocrinol. 2021;94(3):473‐483. doi:10.1111/cen.14313 32799342

[10] Yorke E . Hyperthyroidism and liver dysfunction: a review of a common comorbidity. Clin Med Insights Endocrinol Diabetes. 2022;15: Published 2022 Feb 7. doi:10.1177/11795514221074672 PMC8829710 35153522

[11] Khemichian S , Fong TL . Hepatic dysfunction in hyperthyroidism. Gastroenterol Hepatol (N Y). 2011;7(5):337‐339. 21857837 PMC3127041

